# Comprehensive Knowledge-Driven AI System for Air Classification Process

**DOI:** 10.3390/ma15010045

**Published:** 2021-12-22

**Authors:** Henryk Otwinowski, Jaroslaw Krzywanski, Dariusz Urbaniak, Tomasz Wylecial, Marcin Sosnowski

**Affiliations:** 1 Faculty of Mechanical Engineering and Computer Science, Czestochowa University of Technology, Armii Krajowej 21, 42-201 Czestochowa, Poland; otwinowski@imc.pcz.pl (H.O.); urbaniak@imc.pcz.czest.pl (D.U.); 2 Faculty of Science and Technology, Jan Dlugosz University in Czestochowa, Armii Krajowej 13/15, 42-200 Czestochowa, Poland; j.krzywanski@ujd.edu.pl; 3 Faculty of Production Engineering and Materials Technology, Czestochowa University of Technology, Armii Krajowej 19, 42-201 Czestochowa, Poland; wylecial@wip.pcz.pl

**Keywords:** classification model, separation control, fuzzy logic, machine learning, artificial intelligence

## Abstract

Air classifier devices have a distinct advantage over other systems used to separate materials. They maximize the mill’s capacity and therefore constitute efficient methods of reducing the energy consumption of crushing and grinding operations. Since improvement in their performance is challenging, the development of an efficient modeling system is of great practical significance. The paper introduces a novel, knowledge-based classification (FLClass) system of bulk materials. A wide range of operating parameters are considered in the study: the mean mass and the Sauter mean diameter of the fed material, classifier rotor speed, working air pressure, and test conducting time. The output variables are the Sauter mean diameter and the cut size of the classification product, as well as the performance of the process. The model was successfully validated against experimental data. The maximum relative error between the measured and predicted data is lower than 9%. The presented fuzzy-logic-based approach allows an optimization study of the process to be conducted. For the considered range of input parameters, the highest performance of the classification process is equal to almost 362 g/min. To the best of our knowledge, this paper is the first one available in open literature dealing with the fuzzy logic approach in modeling the air classification process of bulk materials.

## 1. Introduction

Air classifiers are used to separate materials (fine dry powders) by combining particle size, particle shape, and density. They separate particles using airflow and the physical principles of inertia force, drag force, collision, and gravity, with a high-precision classifying process method. Dry classifying is often a more environmental and economical alternative to wet classifying as no water is used. Air classifiers can be used as a single sizing device in an open circuit where the feed is split into fine and coarse products. These classifiers can also be used in a closed circuit with grinding equipment such as ball mills, rod mills, stirred mills, roller mills, hammer mills, vibration mills or jet mills. In this case, the air classifier is used to select the required size material and return the oversized to the milling system. The use of the air classifier maximizes the mill’s capacity, reduces the mill’s energy consumption, and reduces the production costs [1].

It is essential to develop an efficient method of reducing the energy consumption of crushing and grinding operations. These processes expend more than 50% of the total energy in mineral processing plants [2]. It is also estimated that size reduction accounts for up to 50% of the energy used in mining operations [3]. In comminution, only 1% to 2% of the supplied energy is effectively translated into the creation of new surface areas [4]. The majority of the supplied energy is lost as heat or mechanical energy.

Air classifiers are widely used in the following industrial processes: mining, mineral, power engineering, chemical, cement, ceramics, cosmetics, pharmaceutical, pigments, plastics, food, and others. Flammable and explosive, oxidizable materials can be classified with inert gas shielding.

The best material circulation and precision can be achieved when an air classifier is working with a jet mill [5]. Jet milling is a standard grinding method for high added-value materials. It is mainly used for abrasive or heat-sensitive materials or when the grinding process has to be carried out in ultra-high purity conditions. Jet mills are commonly used to produce particles from 1 μm to 10 μm in the chemical, pharmaceutical, and mineral industries. The breakage of particles in the jet mill is dependent on the following operational parameters: classifier rotational speed, feed rate, and grinding pressure. The air classifier has a crucial influence on reducing energy consumption and reducing the grinding costs in a jet mill.

The classification process is widely employed in various technologies. In the literature, there are works on both the theoretical and experimental research of the classification process. In modeling the classification process, numerical methods are often used. Most works use computational fluid dynamics (CFD). Huang et al. (2012) performed inner flow field simulations with Fluent software of a modified turbo air classifier [6]. Material classification performance experiments confirmed the computational fluid dynamics simulation results. The Fluent CFD code was also applied by Guizani et al. (2014) to model the highly turbulent fluid flow and selectivity curves inside a dynamic rotor classifier [7]. The simulation results were analyzed to understand the fish-hook effect and the classifier’s separation mechanism. Liu et al. (2015) used Fluent software to simulate the inner flow of different structures in a turbo air classifier [8]. Calcium carbonate classification experiments were performed to verify the simulation results. A new parametric prediction model of the turbo air classifier cut size was presented by Yu and Liu (2018) in [9]. The inner flow field and Lagrangian equation of particle motion, as well as the particle trajectory in the annular region, were simulated using MATLAB Software. Talc powder classification experiments were carried out to verify this cut size prediction model. Yu et al. (2019) employed a logarithmic spiral volute design method for the turbo air classifier [10]. The Ansys Fluent simulations of airflow motion and discrete phase indicate that the presented method can provide a well-distributed flow field for classification. Zeng et al. (2020) analyzed the influence of the rotor cage speed and inlet air velocity on the flow field in a turbo air classifier using Ansys Fluent Software [11]. Classification experiments of two materials (barite and iron-ore powder) were employed to verify the optimal process parameters. The effects of other parameters as the rotor cage’s outer and the inner radii on the turbo air classifier’s flow field were also analyzed via CFD simulation using Ansys Fluent by Yu et al. (2020) [12]. Calcium carbonate classification experiments were performed. The experimental results reflect the characteristics of the numerically simulated inner flow field in the classifier. The inclined plane classifier, designed for the classification of limestone particles, was modeled and optimized by Petit et al. (2020) in [13]. The velocity and pressure fields inside the classifier were modeled using computational fluid dynamics. The particle trajectories were computed using Lagrangian discrete phase modeling. The Taguchi method was used to optimize the classification performance and the particle size distribution of the classification product.

Apart from the use of CFDs in modeling the classification process, there are works based on Whiten’s approach (in open and closed circuits). Whiten’s efficiency curve approach was used in the mathematical model for high-efficiency air classifiers operating in cement grinding circuits [14]. The variation in the rotor size and air volume parameters with the capacity of the classification process were investigated. Experimental studies of the air classification of materials with different densities (clinker, copper ore, magnetite, coal) were carried out by Altun et al. (2016) [15]. The resulting correlations were integrated into an existing air classifier model. In the presented model, mass balancing studies were performed, and the size-by-size efficiencies were calculated and then put into Whiten’s efficiency curve equation. The classification efficiency of the static air classifier in a vertical spindle mill was investigated by Li et al. (2019) [16]. Samples of the following materials with different particle sizes and densities were used: pyrite, carborundum, quartz, and coal. Whiten’s model was applied to determine the influence of density on the accuracy of classification, cut size, and fish-hook effect. A new model containing both material size and density was established to illustrate the difference in the classification effect of multi-component particles within the classifier.

For the mill’s classifier device, several other models for classification were developed as well. Özer et al. (2010), Özer et al. (2016), Wei et al. (2014), Shi et al. (2015), Kojovic et al. (2015), and Li et al. (2018) investigated classifier parameters empirically [17,18,19,20,21,22]. Classification tests of coal samples were carried out in a static classifier of a vertical spindle mill to investigate the effect of size and density on particle segregation [15,16,17,18,19,20].

Currently, the fuzzy inference approach is increasingly commonly used in modeling various technological processes. The fuzzy method (such as fuzzy artificial neural networks, fuzzy genetic algorithms, fuzzy ant colony optimization, fuzzy artificial immune systems) is an alternative to traditional notions of set membership and logic. Fuzzy inference systems are associated with several names, such as fuzzy-rule-based systems, fuzzy expert systems, fuzzy logic controller, fuzzy model, fuzzy associative memory, and fuzzy system [23,24,25,26].

In modeling the processes of the mechanical processing of mineral raw materials, the fuzzy logic algorithm is most often used in modeling closed milling circuits. A fuzzy-prediction controller was applied to control the overflow density of a milling-classifier’s operating system, which had uncertainty factors and nonlinear, time-delay characteristics in [27]. Practical production has proved that the ore feeding of the ball mill improved significantly. Costea et al. (2015) described a control system architecture for cement milling based on fuzzy logic to adjust the fresh feed [28]. The dynamic behavior of the ball cement mill was simulated using a Matlab Simulink scheme. The modeling of a cement mill was also conducted by Retnam et al. (2016), and fuzzy control was also introduced [29]. The milling system was also simulated using Matlab Simulink. Zhang et al. (2016) employed intelligent fuzzy logic for grinding and classification control. Three grinding-classification circuits were studied [30].

The fuzzy logic approach is rarely used to model the classification process. Yu and Liu (2013) used a turbo air classifier as the classification system and talc powders as the materials [31]. The fuzzy analytic hierarchy process was applied to calculate the weights of the classification performance indices. This assessment method avoids the limitation of evaluating a single classification performance index and incomplete information derived from single-factor experiments. A fuzzy model was developed to predict the cut size of the classifier as a process response by Khoshdast et al. (2019) [32]. The proposed modeling approach was verified by simulating a coal hydraulic classifier in an industrial environment.

The first fuzzy logic-based modeling of a fluidized bed jet milling process is presented in [33]. The following input variables were considered in the study: working air pressure, classifier rotor speed, and test conducting time. The mass of the product and the Sauter mean diameters of the grinding product were the outputs. The results evaluated using the developed FLMillPlus model were in good agreement with the relevant experimental data. The maximum relative errors were lower than 10% [33].

Contemporary trends in the modeling of multiphase systems in mineral processing were presented in Cisternas et al. (2020). Several examples of the applications of CFD in classification were given.

The above literature review shows that the fuzzy-logic approach is rarely used in classification process modeling. The fuzzy-logic approach is one of the paper’s main contributions. Moreover, FL provides a convenient way to map the input to an output space as a precise logic of imprecision and approximate reasoning [34]. Finally, the most crucial advantage of FL-based systems is their ability to perform simple, cheap, and fast solutions when modeling complex systems [23]. The present work aims to develop a comprehensive knowledge-driven AI system to model the material air classification process. Based on previous experience, we developed a fuzzy-logic-based classification (FLClass) system of bulk materials, comprehensively describing the classification process using a wide range of operational variables driving the process.

## 2. Materials and Methods

### 2.1. Description of the Process

**Figure 1:**
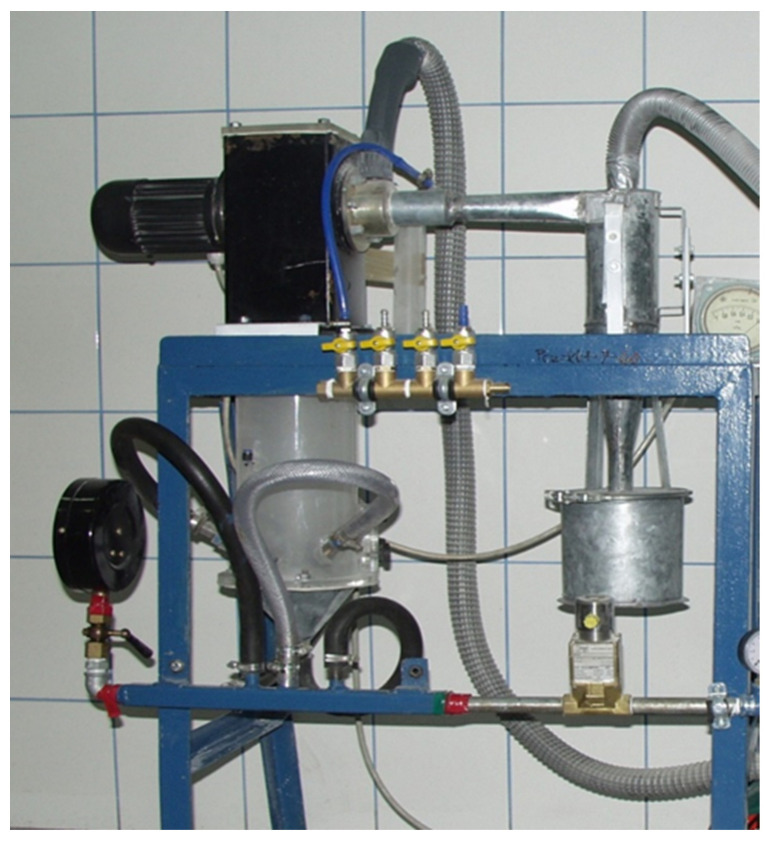
Experimental stand.

**Figure 2:**
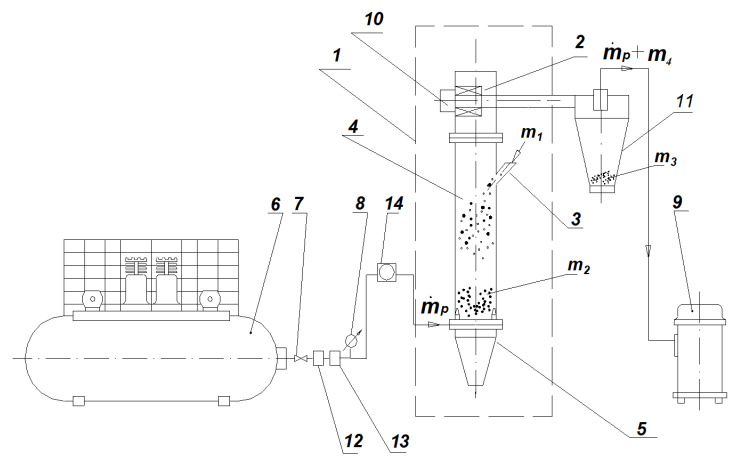
Scheme of experimental stand [35]. 1—turbo air classifier, 2—upper part of classifier chamber with a rotor, 3—feed inlet, 4—cylindrical part of classifier chamber, 5—conical part of classifier chamber, 6—piston compressor with expansion tank, 7—pressure reducing valve, 8—manometer, 9—vacuum cleaner, 10—electric motor, 11—cyclone, 12—air-oil separator, 13—compressed air dryer, 14—flowmeter, *m*_1_—feed mass, *m*_2_—coarse product mass, *m*_3_—fine product mass from the cyclone, *m*_4_—fine product mass from the filter, ${\dot{m}}_{p}$—air mass flux.

The study presented in this paper was conducted on the experimental stand presented in Figure 1. A schematic diagram of the experimental stand is shown in Figure 2.

The system is located at the Faculty of Mechanical Engineering and Computer Science, Czestochowa University of Technology, Poland. Quartz sand was used in the study of the classification process. It is characterized by abrasion resistance; the shape of the sand particles is close to a ball with a density equal to 2638 kg/m^3^, and it corresponds to materials that are the most often applied in the processing industry of mineral raw materials. Low transient humidity is also an important feature. 

Samples of the fed material were fed gravitationally from the feed inlet (3) into the classifier column (4). Working air was entered into the bottom part of the column by four convergent nozzles from the piston compressor (6). After compression, the air underwent treatment in the air-oil separator (12) and dehydrator (13). The overpressure and mass flow of the working air were measured by the elastic pressure gauge (8) and electromagnetic flowmeter (14). The turbo air classifier with a horizontal cylindrical rotor with radial blades was placed over the column (4). 

The rotational speed of the rotor was regulated by an inverter. Particles smaller than the cut size entered the cyclone (11) between the rotor blades. To generate negative pressure in the classifier chamber, a vacuum cleaner (9) was applied. After every test, the classification products were weighed using electronic laboratory scales. The measurements of the particle size distributions of the fed material and classification products were carried out using a KAMIKA Instruments infrared particle sizer. The tested classifier consisted of two parts: the cylindrical part (4) and the upper part with a horizontal rotor (2). The first part accounted for the gravitational stage of the classifier, while the second had the centrifugal stage. Particle distribution is the result of the interaction between these two stages of classification. Determination of the cut size during the two-stage classification was performed using the matrix algorithm and experimental data [35]. 

Two series of experiments were conducted. In the first series of tests, samples of quartz sand with the Sauter mean diameter of 49.8 µm were used. The influence of the classifier operation time on the intensity of the classification process was investigated at different values of compressed air pressure as well as different rotational speeds of the classifier rotor. During the experiments, the working air pressure was changed in the range *p* = 300–700 kPa. The classifier rotor speed was *n* = 25; 50; 75 1/s, and the duration of a single classification test was t = 0.5; 1; 3; 6 min. Longer test times were associated with the risk of grinding the material, which would change the conditions of the classification process. As the pressure increases, the amount of energy fed into the classification process rises. When the tests were carried out at pressures greater than or equal to 500 kPa, the maximum test time was reduced to 3 min in order to prevent the grinding of the classified particles. In the first series of tests, the mass of the samples of the fed material was unchanged, m = 0.5 kg.

In the second series, the samples of quartz sand with the Sauter mean diameter of 46.5 µm were tested. The pressure of the working air was changed over the range of *p* = 100–600 kPa during the experiments. The classifier rotor speed was *n* = 0; 7.5; 15; 25 1/s. During the tests the static values were the mass of the fed material m = 1 kg and the time of the classification process t = 3 min. Due to the nature of the experiments, the feed mass was not related to the duration of the experiment. The test results are presented in Table 1 (first series) and Table 2 (second series). Four values of the Sauter diameter are not indicated in Table 2, because for these working parameters all the feed material was directed to the coarse product. Table 3 shows the results of the experiments that were used to validate the model. In these tests, samples of quartz sand with the Sauter mean diameter of 49.8 µm were used and the mass of the fed material was equal to m = 0.5 kg. The rotational speed of the classifier rotor was equal to *n* = 37.5 and 50 1/s, the working air pressure was changed in the range *p* = 300–700 kPa, and the duration of a single classification test was t = 2; 3; 4 min.

### 2.2. Modeling of the Classification Process

The fuzzy-logic-based modeling approach belongs to so-called soft-computing methods [36,37,38,39,40,41]. The technique was introduced in 1965 by Lofti Zadeh, who defined it as a precise logic of imprecision and approximate reasoning [34,42,43,44]. It is now one of the most popular, knowledge-based artificial intelligence (AI) methods used in cases when subjective expert knowledge is essential in defining the objective function and decisive variable [23,24,43,44,45,46]. It is an effective way of mapping an input domain into the output domain. The method is based on fuzzy sets and membership functions, which define how each input variable is mapped to a membership value between 0 and 1 [23]. Two types of fuzzy inference systems are used: the Mamdami type, where the membership function is a fuzzy set, and the Sugeno type (sometimes called the TSK models or Takagi, Sugeno, and Kang models), where the output is a polynomial function [24]. A fuzzy-logic-based model consists of the following main components: a fuzzifier, a fuzzy rule base, an inference engine, and a defuzzifier covering the fuzzification, inference, and defuzzification operations. A further detailed description of the fuzzy-logic-based method can be found elsewhere [23,24,33,47].

The developed FLClass system considers a wide range of input and output variables. The following five input variables were selected to develop the proposed comprehensive FLClass material classification system: mass and the Sauter mean diameter of the fed material (mf and daf, respectively), rotational speed n of the classifier rotor, pressure p of the working air, and time t of conducting the test. Performance g (the fine product mass flow), Sauter mean diameter dap, and cut size X of the classification product constitute the output variables. The inputs and outputs are described in Table 4.

**Figure 3:**
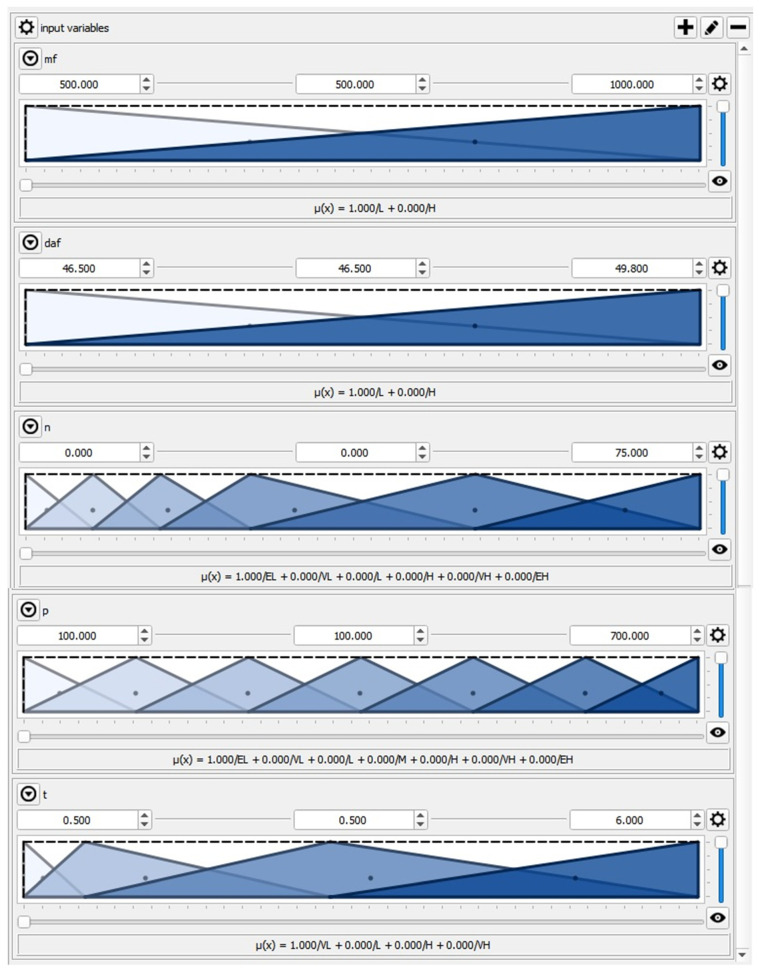
Membership functions for input variables: m_f_, d_af_, n, p, t (x-axes and y-axes correspond to parameter values from Table 4 and values of membership function, respectively).

The QtFuzzyLite fuzzy logic control application, ver. 5.5.1, by the Qt Company Ltd., Wellington, New Zealand [48] was used in the presented model research. The five input features were covered by triangular linguistic variables, according to Figure 3.

**Figure 4:**
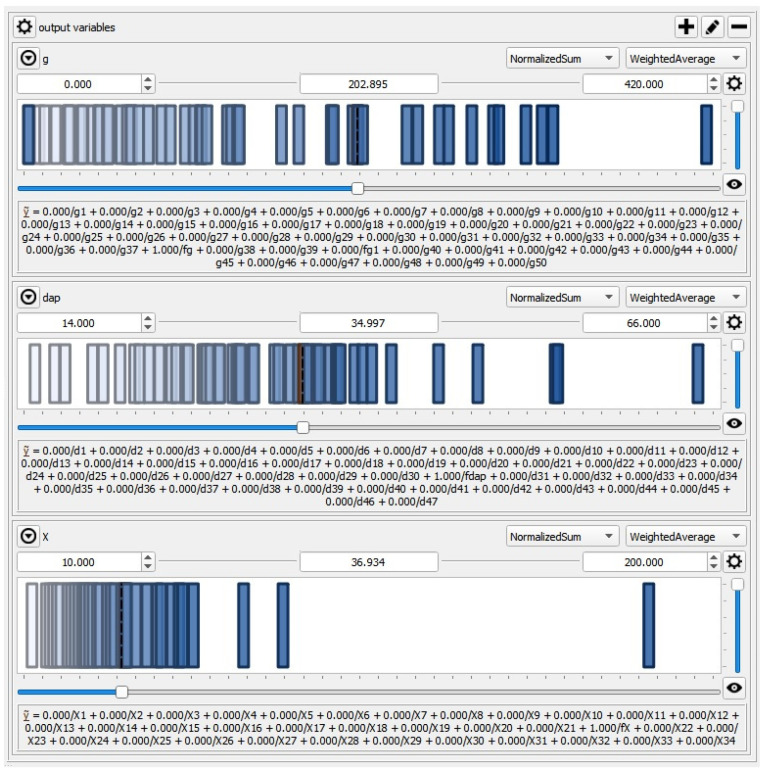
Membership functions for output parameters g, d_ap_ and X (x-axes and y-axes correspond to parameter values from Table 4 and values of membership function, respectively).

The Takagi–Sugeno inference engine is used in the FLClass model. Constant and polynomial linguistic terms are used to accurately describe the output variables, as depicted in Figure 4.

The set of fuzzy IF-THEN rules, allowing the model to be expressed, are formulated and summarized in Table 5.

The IF criteria belong to the inputs, while the THEN criteria belong to the output features [23]. Finally, the weighted average method is employed during the defuzzification stage. This final operation leads to the generation of crisp outputs as an answer to crisp inputs [24,33].

Such a developed robust FLClass system allows the air classification process to be described based on the expertise of human experts. This knowledge-based system provides an alternative modeling approach, considering the complexity and high costs of the other methods of data handling [23,49,50].

## 3. Results and Discussion

**Figure 5:**
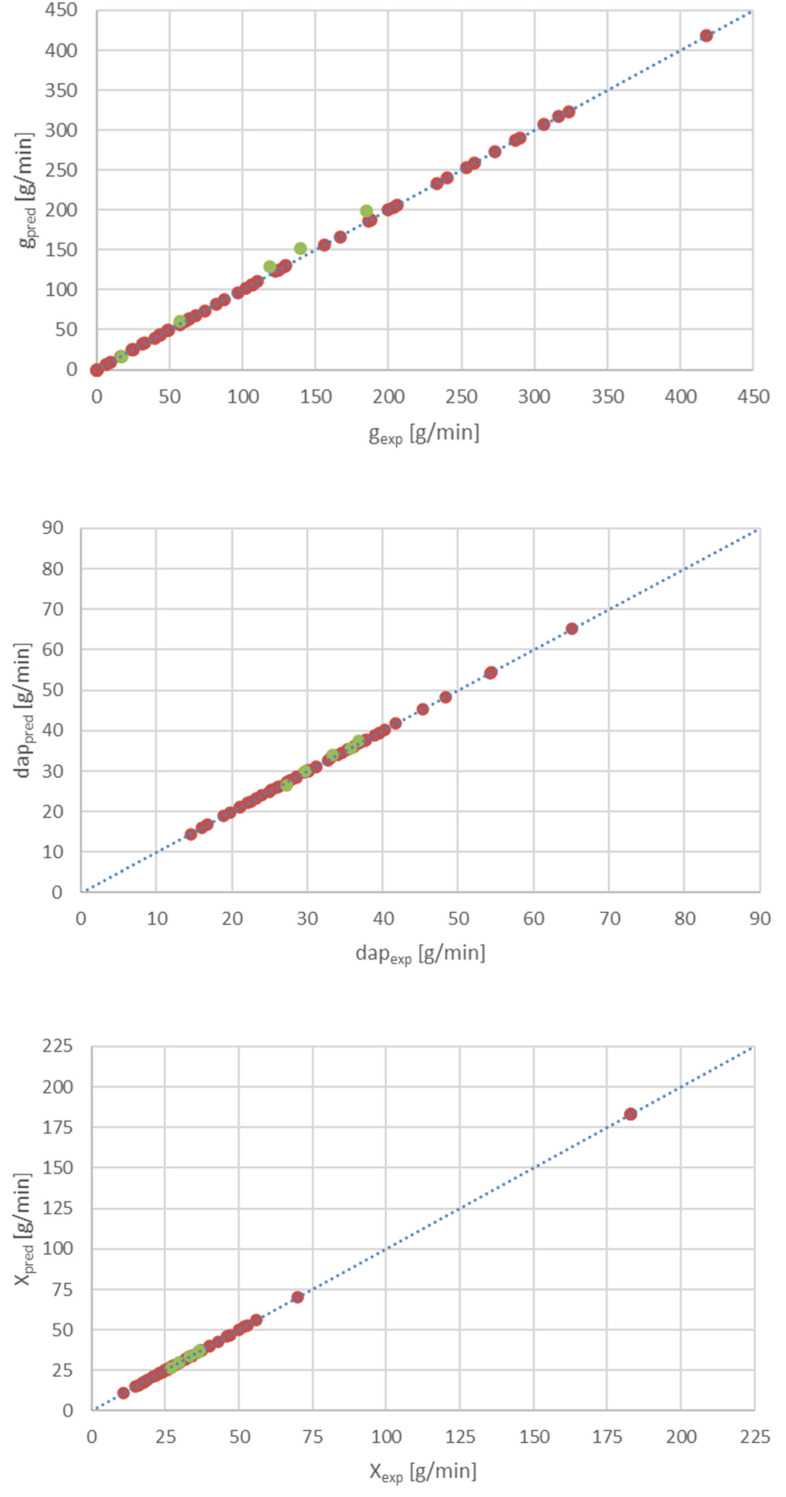
Comparison of desired g, d_ap_ and X (measured, from experiment) that and predicted by FLClass model (brown symbols 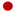 refer to data used to build the model, while green ones 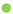 apply to a new testing data set, previously unseen by the system).

The FLClass system was successfully validated against the experimental results unseen by the model. These data were not previously used in the development process of the model. The maximum relative errors between the measured and calculated data for g, d_ap_, and X are lower than 9% (Figure 5).

Good performance of the developed FLClassSystem was achieved, even for the new testing data set. The predicted results are located within the range of ±9%, compared to the experimental data. Such a small relative error forms a solid basis for the possibility of using the developed model in practice.

**Figure 6:**
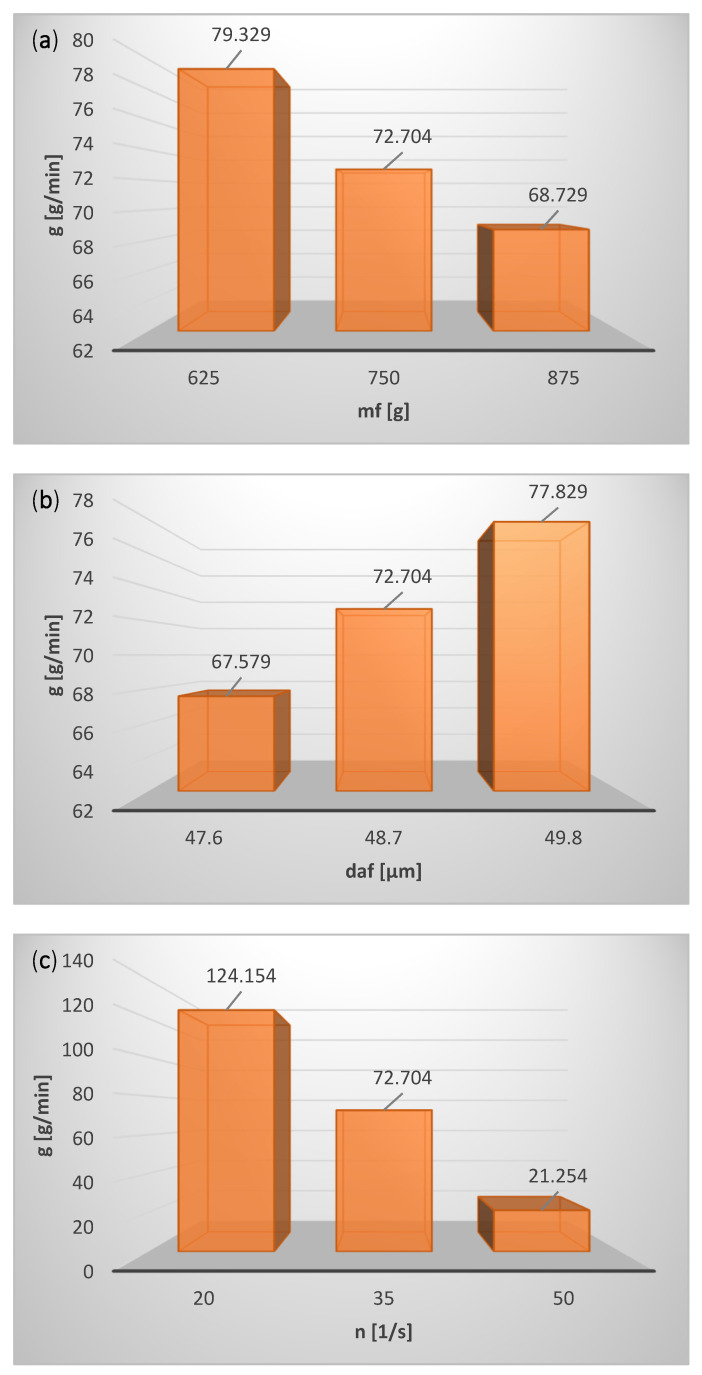

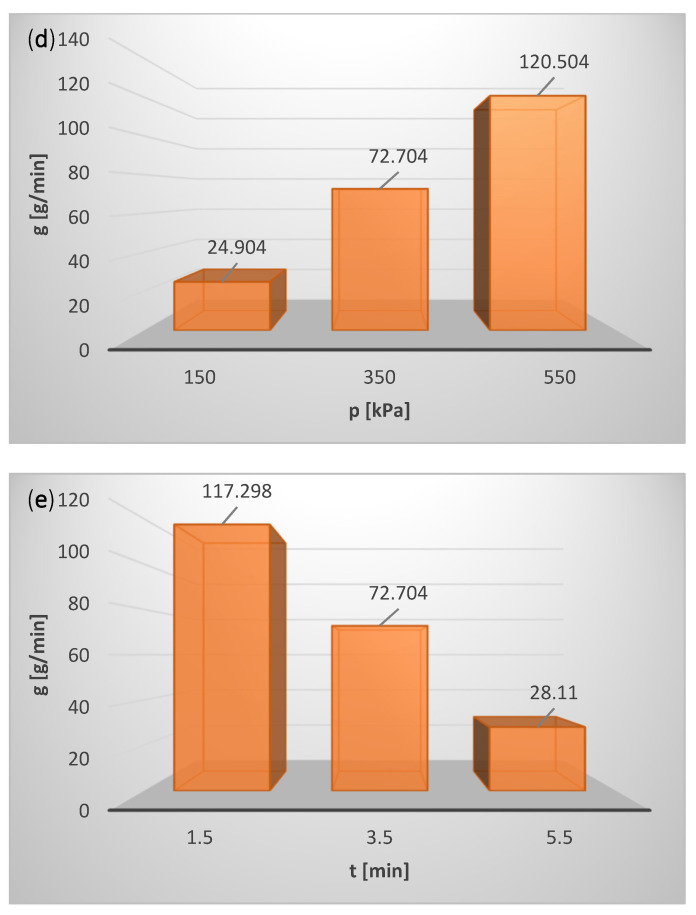
Influence of operating parameters on performance of classification process for (**a**) mass of fed material, m_f_, (**b**) Sauter mean diameter of fed material, d_af_, (**c**) classifier rotor speed, n, (**d**) working air pressure, p, (**e**) test conducting time, t.

The influence of the operating parameters on the performance of the classification process is shown in Figure 6.

**Figure 7:**
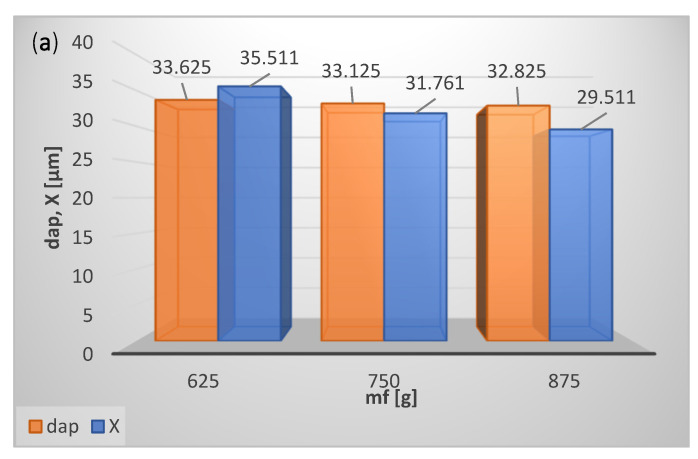

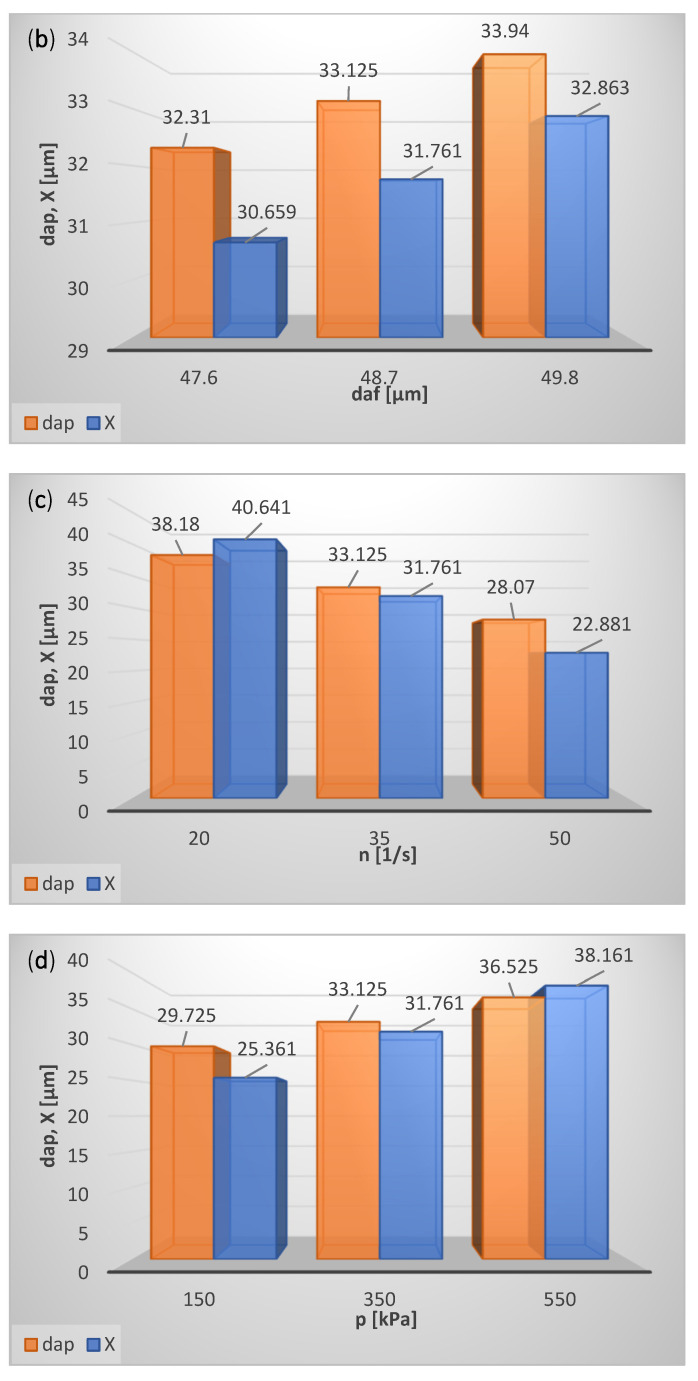

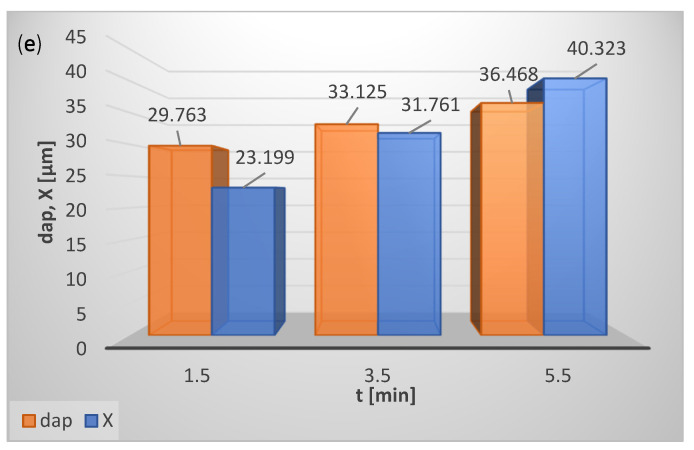
Influence of operating parameters on performance g of classification process and cut size X of product for (**a**) mass of fed material, m_f_, (**b**) Sauter mean diameter of fed material, d_af_, (**c**) classifier rotor speed, n, (**d**) working air pressure, p, (**e**) test conducting time, t.

The effects of the input variables on Sauter mean diameter d_ap_ of the product and cut size X are depicted in Figure 7.

In the studied range of variability of the classification process parameters and the particle size distribution of the feed, based on the calculation results the following detailed conclusions can be formulated:

With the increase in the mass of the feed, m_f_, the material concentration in the classification zone rises, as a result of which classifier performance g decreases (Figure 6a), and cut size X as well as Sauter mean diameter d_ap_ of the classification product decrease (Figure 7a). A reduction of the classifier performance g with an increase in m_f_ may result from the two-stage nature of the classification process, and it certainly requires further research.

Classifier performance g (Figure 6b), cut size X as well as Sauter mean diameter of the product dap (Figure 7b) grow with the increase in the feed particle size (Sauter mean diameter d_af_) because the fraction of coarse particles in the classification product increases.

With the increase in rotational speed of the classifier rotor n, classifier performance g decreases (Figure 6c) due to the fall in cut size X and Sauter mean diameter d_ap_ of the classification product (Figure 7c). 

As the working air pressure rises, the air mass flow grows, carrying the coarse particles to the fine product, which increases classifier performance g (Figure 6d), cut size X, and Sauter mean diameter d_ap_ of the classification product (Figure 7d).

With the increase in time (with the passing of time), the particle concentration in the classification zone decreases, and classifier performance g declines (Figure 6e). In the initial phase of classification, first the fine particles are separated, which results in an increase in the average particle size of the material remaining in the fluidized bed; this material in the next phase of classification goes to the fine product (Sauter mean diameter of the product d_ap_ and cut size X increase) (Figure 7e).

## 4. Best Strategy in the Classification Process

Considering the observed trends in the performance behavior, an impression of the effects of the input parameters on g can be described as shown in Table 6.

As we can see, the performance of the classification process can be enhanced by the decrease in mass of the fed material, classifier rotor speed, and shortening of the test duration time. The classification process can achieve further performance improvement by increasing the working air pressure and the Sauter mean diameter of the feed material. Therefore, for the considered range of input parameters, the highest performance g can be attained for the following conditions: mass of the fed material, m_f_ = 500 g, Sauter mean diameter of the fed material d_af_ = 49.8 µm, classifier rotor speed, *n* = 0, s^−1^, working air pressure *p* = 700 kPa and test conducting time, t = 0.5 min. 

The highest value of g, which can be acquired for the considered range of input operational parameters, is equal to 361.67 g/min.

The model developed in the paper has a universal character as it uses inputs independent of the type and size of classifiers and material used. However, since the model was performed and validated on the specific conditions described in the paper, additional inputs relating to materials properties, such as density or/and particles sphericity, may be necessary to separate different combinations of materials and achieve reasonable accuracy.

## 5. Conclusions

The paper introduces a novel, knowledge-based classification (FLClass) system of bulk materials. The model was successfully validated against experimental data. The maximum relative error between the measured and predicted data is lower than 9%.

The comprehensive system considers a wide range of operating parameters, i.e., mean mass of the fed material, the Sauter mean diameter of the fed material, classifier rotor speed, working air pressure, and test conducting time. 

The developed model can predict the Sauter mean diameter and the cut size of the classification product, as well as the performance of the process.

The presented fuzzy-logic-based approach allows an optimization study to be conducted of the process.

The highest value of g that can be obtained for the considered range of input operational parameters is equal to 361.67 g/min.

To the best of our knowledge, this paper is the first one available in open literature dealing with the use of the fuzzy logic method in the modeling of the air classification process of bulk materials.

## Figures and Tables

**Table 1:** Performance values, Sauter mean diameter of fine product and cut size for different test times, for different rotational speeds of classifier rotor, and for different pressures of working air (first series).

Working Air Pressure	Classifier Rotor Speed	Test Time	Performance	Sauter Mean Diameter of Fine Product	Cut Size
p, kPa	n, 1/s	t, min	g, g/min	d_ap_, µm	X, µm
300	25	0.5	187.00	27.3	26
300	25	1	129.00	34.0	37.5
300	25	3	68.00	35.4	46
300	25	6	43.23	37.0	52
300	50	0.5	130.00	21.0	18
300	50	1	87.50	26.0	26
300	50	3	39.67	28.6	32
300	50	6	24.58	30.2	36
300	75	0.5	61.00	16.0	11
300	75	1	44.00	21.0	17
300	75	3	24.33	22.5	23
300	75	6	16.92	24.0	26
500	25	0.5	290.00	28.5	32
500	25	1	188.00	36.0	40
500	25	3	106.00	37.5	50
500	50	0.5	156.00	23.2	23
500	50	1	102.00	31.2	30
500	50	3	49.33	33.1	34
500	75	0.5	74.00	19.7	15
500	75	1	57.00	27.6	22
500	75	3	25.33	30.1	26
700	25	0.5	418.00	30.1	37
700	25	1	259.00	38.9	47
700	25	3	122.67	40.2	53
700	50	0.5	206.00	25.3	27
700	50	1	125.00	34.6	34
700	50	3	63.33	36.2	37
700	75	0.5	110.00	22.1	19
700	75	1	82.00	29.6	25
700	75	3	48.67	31.1	29.5

**Table 2:** Performance values, Sauter mean diameter of fine product and cut size for different rotational speeds of classifier rotor, and for different pressures of working air (second series).

Working Air Pressure	Classifier Rotor Speed	Test Time	Performance	Sauter Mean Diameter of Fine Product	Cut Size
p, kPa	n, 1/s	t, min	g, g/min	d_ap_, µm	X, µm
100	0		203.30	45.3	28
200	0	3	233.30	48.3	33
300	0	3	253.67	54.2	40
400	0	3	286.67	65.1	70
500	0	3	306.67	-	183
600	0	3	323.33	-	183
100	7.5	3	31.67	37.6	25.5
200	7.5	3	96.67	39.5	28
300	7.5	3	166.67	41.7	34
400	7.5	3	200.00	54.4	56
500	7.5	3	273.33	-	81
600	7.5	3	316.67	-	200
100	15	3	0.00	27.9	23
200	15	3	0.00	29.9	25
300	15	3	6.67	32.8	27
400	15	3	106.67	35.3	28
500	15	3	186.67	37.8	33
600	15	3	240.00	39.5	43
100	25	3	0.00	14.5	16
200	25	3	0.00	16.8	18
300	25	3	0.00	18.9	21
400	25	3	10.00	24.9	23
500	25	3	33.33	26.2	24
600	25	3	63.33	27.4	26

**Table 3:** Performance values, Sauter mean diameter of fine product and cut size for different test times, for different rotational speeds of classifier rotor, and for different pressures of working air (validation data).

Working Air Pressure	Classifier Rotor Speed	Test Time	Performance	Sauter Mean Diameter of Fine Product	Cut Size
p, kPa	n, 1/s	t, min	g, g/min	d_ap_, µm	X, µm
300	50	2	52.25	27.2	30
300	50	4	32.50	29.7	35
500	37.5	2	81.00	33.4	35
500	37.5	3	73.33	35.1	37
700	37.5	2	124.00	36.8	45

**Table 4:** Model variables.

Variables	Values
Inputs	
Mass of fed material, mf, g	500–1000
Sauter mean diameter of fed material, d_af_, µm	46.5–49.8
Classifier rotor speed, n, s^−1^	0–75
Working air pressure, p, kPa	100–700
Test conducting time, t, min	0.5–6
Outputs	
Performance, g, g/min	0–418
Sauter mean diameter of classification product, d_ap_, µm	14.5–65.1
Cut size of classification product, X, µm	11–183

**Table 5:** Fuzzy rule base of developed FLClass system.

ID	Rule
1	if mf is L * and daf is H and n is EH and p is L and t is VL then g is g16 and dap is d2 and X is X1
2	if mf is L and daf is H and n is EH and p is H and t is VL then g is g20 and dap is d5 and X is X2
3	if mf is H and daf is L and n is H and p is EL and t is H then g is g1 and dap is d1 and X is X3
4	if mf is L and daf is H and n is EH and p is L and t is L then g is g12 and dap is d6 and X is X4
5	if mf is H and daf is L and n is H and p is VL and t is H then g is g1 and dap is d3 and X is X5
6	if mf is L and daf is H and n is VH and p is L and t is VL then g is g31 and dap is d6 and X is X5
7	if mf is L and daf is H and n is EH and p is EH and t is VL then g is g27 and dap is d7 and X is X6
8	if mf is H and daf is L and n is H and p is L and t is H then g is g1 and dap is d4 and X is X7
9	if mf is L and daf is H and n is EH and p is H and t is L then g is g15 and dap is d17 and X is X8
10	if mf is L and daf is H and n is EH and p is L and t is H then g is g5 and dap is d8 and X is X9
11	if mf is L and daf is H and n is VH and p is H and t is VL then g is g32 and dap is d9 and X is X9
12	if mf is H and daf is L and n is H and p is M and t is H then g is g3 and dap is d11 and X is X9
13	if mf is H and daf is L and n is L and p is EL and t is H then g is g1 and dap is d18 and X is X9
14	if mf is H and daf is L and n is H and p is H and t is H then g is g9 and dap is d14 and X is X10
15	if mf is L and daf is H and n is EH and p is EH and t is L then g is g21 and dap is d21 and X is X11
16	if mf is H and daf is L and n is L and p is VL and t is H then g is g1 and dap is d22 and X is X11
17	if mf is H and daf is L and n is VL and p is EL and t is H then g is g8 and dap is d37 and X is X12
18	if mf is L and daf is H and n is EH and p is L and t is VH then g is g4 and dap is d10 and X is X13
19	if mf is L and daf is H and n is VH and p is L and t is L then g is g22 and dap is d13 and X is X13
20	if mf is L and daf is H and n is H and p is L and t is VL then g is g35 and dap is d15 and X is X13
21	if mf is H and daf is L and n is H and p is VH and t is H then g is g18 and dap is d16 and X is X13
22	if mf is L and daf is H and n is EH and p is H and t is H then g is g7 and dap is d23 and X is X13
23	if mf is L and daf is H and n is VH and p is EH and t is VL then g is g39 and dap is d12 and X is X14
24	if mf is H and daf is L and n is L and p is L and t is H then g is g2 and dap is d27 and X is X14
25	if mf is H and daf is L and n is L and p is M and t is H then g is g26 and dap is d31 and X is X15
26	if mf is H and daf is L and n is VL and p is VL and t is H then g is g23 and dap is d40 and X is X15
27	if mf is H and daf is L and n is EL and p is EL and t is H then g is g38 and dap is d43 and X is X15
28	if mf is L and daf is H and n is EH and p is EH and t is H then g is g13 and dap is d25 and X is X16
29	if mf is L and daf is H and n is VH and p is H and t is L then g is g24 and dap is d26 and X is X17
30	if mf is L and daf is H and n is H and p is H and t is VL then g is g46 and dap is d19 and X is X18
31	if mf is L and daf is H and n is VH and p is L and t is H then g is g10 and dap is d20 and X is X18
32	if mf is H and daf is L and n is L and p is H and t is H then g is g34 and dap is d38 and X is X19
33	if mf is H and daf is L and n is EL and p is VL and t is H then g is g40 and dap is d44 and X is X19
34	if mf is L and daf is H and n is VH and p is H and t is H then g is g14 and dap is d28 and X is X20
35	if mf is L and daf is H and n is VH and p is EH and t is L then g is g29 and dap is d30 and X is X20
36	if mf is H and daf is L and n is VL and p is L and t is H then g is g33 and dap is d42 and X is X20
37	if mf is L and daf is H and n is VH and p is L and t is VH then g is g6 and dap is d24 and X is X21
38	if mf is L and daf is H and n is H and p is EH and t is VL then g is g50 and dap is d23 and X is X22
39	if mf is L and daf is H and n is VH and p is EH and t is H then g is g17 and dap is d34 and X is X22
40	if mf is L and daf is H and n is H and p is L and t is L then g is g30 and dap is d29 and X is X23
41	if mf is L and daf is H and n is H and p is H and t is L then g is g36 and dap is d33 and X is X24
42	if mf is H and daf is L and n is EL and p is L and t is H then g is g42 and dap is d45 and X is X24
43	if mf is H and daf is L and n is L and p is VH and t is H then g is g41 and dap is d40 and X is X25
44	if mf is L and daf is H and n is H and p is L and t is H then g is g19 and dap is d32 and X is X26
45	if mf is L and daf is H and n is H and p is EH and t is L then g is g43 and dap is d39 and X is X27
46	if mf is L and daf is H and n is H and p is H and t is H then g is g25 and dap is d36 and X is X28
47	if mf is L and daf is H and n is H and p is L and t is VH then g is g11 and dap is d35 and X is X29
48	if mf is L and daf is H and n is H and p is EH and t is H then g is g28 and dap is d41 and X is X30
49	if mf is H and daf is L and n is VL and p is M and t is H then g is g37 and dap is d46 and X is X31
50	if mf is H and daf is L and n is EL and p is M and t is H then g is g45 and dap is d47 and X is X32
51	if mf is H and daf is L and n is VL and p is H and t is H then g is g44 and X is X33
52	if mf is H and daf is L and n is EL and p is H and t is H then g is g47 and X is X34
53	if mf is H and daf is L and n is VL and p is VH and t is H then g is g48 and X is X34
54	if mf is H and daf is L and n is EL and p is VH and t is H then g is g49 and X is X34
55	if mf is any and daf is any and n is any and p is any and t is any then g is fg and dap is fdap and X is fX

* EL—extremely low, VL—very low, L—low, M—medium, H—high, VH—very high, EH—extremely high.

**Table 6:** Effect of increase in input parameters on performance g of classification process.

Parameter (Horizontal Axis)	g (Vertical Axis)
Mass of fed material, mf, g	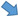
Sauter mean diameter of fed material, d_af_, µm	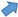
Classifier rotor speed, n, s^−1^	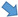
Working air pressure, p, kPa	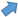
Test conducting time, t, min	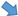
